# Gut microbiota-derived fatty acid and sterol metabolites: biotransformation and immunomodulatory functions

**DOI:** 10.1080/19490976.2024.2382336

**Published:** 2024-07-24

**Authors:** Haohao Zhang, Yadong Xie, Fei Cao, Xinyang Song

**Affiliations:** Key Laboratory of Multi-Cell Systems, Shanghai Institute of Biochemistry and Cell Biology, Center for Excellence in Molecular Cell Science, Chinese Academy of Sciences, University of Chinese Academy of Sciences, Shanghai, China

**Keywords:** Gut microbiota, fatty acids, steroids, biotransformation, immunity

## Abstract

Commensal microorganisms in the human gut produce numerous metabolites by using small molecules derived from the host or diet as precursors. Host or dietary lipid molecules are involved in energy metabolism and maintaining the structural integrity of cell membranes. Notably, gut microbes can convert these lipids into bioactive signaling molecules through their biotransformation and synthesis pathways. These microbiota-derived lipid metabolites can affect host physiology by influencing the body’s immune and metabolic processes. This review aims to summarize recent advances in the microbial transformation and host immunomodulatory functions of these lipid metabolites, with a special focus on fatty acids and steroids produced by our gut microbiota.

## 1. Introduction

Lipid molecules are an important source of energy for the human body and are also essential for maintaining the structural stability of cell membranes. At the same time, the structural diversity of lipid molecules determines their function as signaling molecules involved in many physiological and pathological processes in the body. Lipid molecules are not only derived from the diet or the host’s metabolic pathways but can also be synthesized and converted by the commensal microbial communities that colonize the intestinal mucosal surface. In recent years, the role of lipid molecules derived from symbiotic microorganisms in maintaining human health or in the progression of disease has attracted widespread research interest.

The intestinal mucosal immune system consists of a single layer of intestinal epithelial cells, innate and adaptive immune cells residing in the intraepithelial or lamina propria region, and immune cells within the gut-associated lymphoid tissues (GALTs) such as Peyer’s patches and mesenteric lymph nodes.^1,2^ Because the intestine is in close contact with external stimuli, the intestinal immune system must defend against foreign pathogens and develop tolerance to commensal microorganisms and food antigens. Thus, intestinal mucosal immune cells are distributed along the intestinal tract with strong regional heterogeneity, and their development and maturation are accompanied by the establishment of a symbiotic relationship between the human body and external microorganisms.^1,2^

Human symbiotic microorganisms include bacteria, fungi, viruses, and a small number of protists that live with us, and the collection of all these microbes and their genes is called the microbiome.^3,4^ A recent study estimated that the gut’s symbiotic microorganisms are dominated by bacteria, with thousands of species, and their number reaches 38 trillion, slightly more than the total number of cells in our body, which is 30 trillion.^5^ Intestinal commensal bacterial species are dominated by 7 phyla: Bacteroidetes, Firmicutes, Proteobacteria, Verrucomicrobia, Actinobacteria, Fusobacteria, and Cyanobacteria.^3^ As the “second genome” of the human body, the biosynthetic/transformational network encoded by the microbiome can convert natural precursors, including lipid molecules, etc., derived from the body or food into a variety of small molecules with host-modulating activities.^6,7^ Bioactive lipid molecules derived from symbiotic bacteria include structural lipid molecules and biotransformed lipid metabolites. The former mainly includes microbial membrane lipid components such as phospholipids, glycerolipids, glycolipids, sphingolipids, and sulfonolipids.^8,9^ The latter consists mainly of microbial lipid metabolites such as bile acids (BAs), cholesterol/hormone derivatives, and various short-chain or long-chain fatty acid (SCFA or LCFA) metabolites.^8,9^

## 2. The lipid diversity of gut microbiota

The structural lipids of symbiotic bacteria are critical for the integrity of their cell membranes, the stability of membrane proteins, the energy production of the electron transport chain, and the resistance to external environmental stresses. Currently, our understanding of microbial membrane lipid molecules comes mainly from intestinal Gram-negative bacterial species such as *Escherichia coli*, *Bacteroides*, *Alistipes*, and *Prevotella*, etc. Similar to the membrane lipid components of eukaryotes, the membrane lipids of symbiotic bacteria also include different types of phospholipid molecules, such as phosphoethanolamine (PE), phosphoglycerol (PG), phosphocholine (PC), phosphoserine (PS), and phosphoinositol (PI), or different types of glycerolipid molecules, such as diacylglycerol (DAG) and triacylglycerol (TAG).^10,11^ Other classes of lipids are restricted to specific bacterial taxa, such as the sphingolipid molecules found in *Bacteroides* strains, including ceramide-phosphoethanolamine (CerPE), ceramide-phosphoinositol (CerPI), dihydroceramide (DHCer), α-galactosylceramide (α-GC), sphinganine, and deoxy sphingolipids.^12^ Gram-negative bacteria also synthesize saccharolipids not found in eukaryotes, such as lipopolysaccharides (LPS) or lipooligosaccharides (LOS), which are composed of acylated lipid groups (Lipid A) and variously sized head groups of sugar structures.^13,14^ Compared to host cell membrane lipids, microbial membrane lipids have unique structural features. For example, the length of their lipid acyl chains can be odd or even, and their ends can form a branched chain structure. The microbial lipid structures differ from those of eukaryotes, making these molecules likely to be recognized by the host’s innate immune system and to activate innate immune sensors such as Toll-like or C-type lectin receptors.^8,9,15–18^ In addition, variations in the structure of microbial lipids, such as LPS or LOS, also determined the immunogenicity of these molecules.^18,19^ However, microbial structural lipids can also exert modulatory functions on the host immune system. For example, the sphingolipids from *Bacteroides fragilis* limited the number of gut-invariant natural killer T cells (iNKT) to prevent the inflammatory effects caused by their hyperactivation.^20,21^

Gut symbiotic microorganisms also convert natural precursors from host or dietary sources into various lipid metabolites, including fatty acids and steroid molecules. Gut microbes can produce a range of SCFAs (the FA chain contains 2–6 carbon atoms). Among them, SCFAs with linear chain structures such as acetic acid, propionic acid, and butyric acid are mainly derived from microbial fermentation of dietary fiber, while SCFAs with branched-chain structures such as 2-methyl-butyric acid, isobutyric acid, and isovaleric acid are mainly derived from the branched-chain amino acid (BCAA) metabolism pathway of microorganisms.^22^ Microbial LCFAs (the FA chain contains more than 12 carbon atoms) have unique structural characteristics and originate from different types of biosynthetic or conversion pathways. For example, several iso- or anteiso-FAs have been synthesized from BCAA-derived α-keto acids, such as iso-15:0 FA or anteiso-15:0 FA.^23,24^ Microbes can also convert monounsaturated FAs to cyclopropane FAs, such as 9,10-methylene hexadecanoic acid, which activates the brain angiogenesis factor 1 (BAI1) receptor in host cells.^23,25^ In addition, intestinal bacteria can convert unsaturated fatty acids, such as linoleic acid (LA) or linolenic acid (LNA), to various FA isomers and their hydroxylated or oxidized intermediates via the LCFA isomerization pathway.^26^ Microbes can also synthesize other long-chain lipid metabolites, such as fatty amide commendamide or serine dipeptide lipids, by forming an amide bond between amines and fatty acids,^27–30^ or convert arachidonic acid into eicosanoids such as prostaglandins.^31^ The microbial sterol lipids are mainly composed of various BA derivatives, including deconjugated BAs, secondary BAs, and newly discovered microbially conjugated BAs,^32–34^ and various types of cholesterol/hormone derivatives, such as sulfonated cholesterol or estrogen, etc.^35–38^

Microbial lipid molecules are associated with the diversity of the microbiome and host health outcomes. For instance, consuming dietary fiber increased gut microbial diversity and SCFA production.^39^ Conversely, dietary fiber deprivation led to a loss of microbiota diversity and impaired host gut barrier functions.^40,41^ As fermentation end products, SCFAs have been shown to have various host modulatory functions. These include enhancing gut barrier function,^42,43^ maintaining gut motility,^44,45^ and controlling gut hormone release.^46–48^ BAs have been reported to be the primary gut steroid molecules responsible for the maturation of the gut microbiota in newborns,^49^ and microbial metabolism of BAs can alter host metabolic phenotypes.^50–52^ In addition to modulating host metabolism, microbial lipids may also serve as immune signaling molecules in the gut mucosa, regulating the fate programming and functionality of different types of intestinal immune cells, as well as host mucosal homeostasis and disease susceptibility. Given the biomass and diversity of human symbiotic microorganisms, research on the biosynthetic pathways and structural identification of microbial lipid molecules is emerging. Here, we summarize recent advances in this field, focusing on the bacterial transformation pathways of various FAs and BAs and the immunomodulatory functions of the resulting metabolites. However, we should appreciate that bacterial structural lipids also interact with host immunological or metabolic pathways, which has been reviewed in detail elsewhere.^10,12,53^

## 3. Microbial transformations of gut fatty acids

### 3.1. Short-chain fatty acids

**Figure 1.**
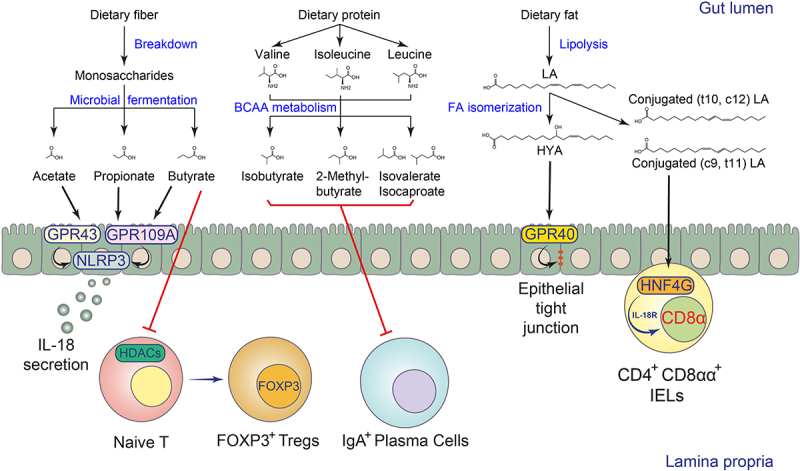
Biotransformation and immunomodulation of microbial FAs in the intestine.

Gut microbes ferment dietary fibers to produce SCFAs, especially acetic, propionic, and butyric acids.^39,54^ The levels of fermentable fibers in human diets may influence the SCFA pool in the gut.^39,54^ Gut microbes can break down the host’s indigestible fibers into monosaccharides such as hexoses, pentoses, fucose, and rhamnose, and then convert them to SCFAs through various enzymatic cascades.^54^ For example, hexoses and pentoses can be metabolized into pyruvate, and one of the representative SCFAs, acetic acid, can be synthesized from pyruvate via either the acetyl-CoA or Wood-Ljungdahl pathway in bacteria. Pyruvate can also be metabolized into lactate or succinate, which are further converted to propionyl-CoA for propionic acid biosynthesis. Gut microbes also ferment deoxyhexose sugars such as fucose or rhamnose into propionic acid via the propanediol pathway. In addition, two molecules of acetyl-CoA can be subsequently reduced into butyryl-CoA for butyric acid biosynthesis. The microbial fermentation of proteins also contributes to the gut SCFA pool via the production of branched-SCFAs such as 2-methyl-butyric acid, isovaleric acid, and isobutyric acid, which are derived from BCAAs isoleucine, leucine, and valine^54^ (Figure 1).

SCFAs are produced by microbial fermentation of dietary fiber. They may promote host gut immunity via GPCR-dependent mechanisms, such as signaling through epithelial GPR43/109A to activate the NLRP3 inflammasome for IL-18 secretion, or via GPCR-independent mechanisms, such as inhibiting HDACs for Treg differentiation. Branched SCFAs are derived from BCAA metabolism and have been shown to inhibit the accumulation of IgA^+^ plasma cells in the gut. Microbial LCFA metabolites, such as LA derivatives, are formed from microbial FA isomerization. The hydroxylated product, HYA, promotes intestinal barrier functions via epithelial GPR40 signaling, while FA isomers, namely CLAs, promote CD4^+^CD8αα^+^ IEL differentiation via HNF4G-instructed IL-18 signaling.

SCFAs can be produced by many types of gut microbes, including members of the genera *Clostridium*, *Eubacterium*, and *Butyrivibrio*.^55^ However, due to the complexity of the microbiome, it remains difficult to elucidate the impact of different microbial SCFA pathways on specific end products. The application of microbial genetics technology can help us understand the impact of fermentation pathways in different microorganisms on SCFA biosynthesis. To this end, Guo *et al*. predicted the potential metabolic gene clusters for small molecule synthesis in *Clostridium sporogenes* and developed a CRISPR-Cas9-based genetic manipulation system for this species to validate key genes responsible for the production of SCFAs as well as other metabolites.^22^ In this study, they confirmed that the production of propionate and butyrate in *Clostridium sporogenes* requires the catabolic threonine dehydratase TdcB and an enoyl-CoA hydratase homolog encoded by the *talA* and *croA* genes, respectively.^22^ Intestinal *Bacteroides* species can also produce SCFAs. For example, in *Bacteroides thetaiotaomicron*, propionate production was mediated by the enzyme methylmalonyl-CoA mutase, which was also common in other enteric *Bacteroides* species.^56^

In the bacterium *Streptomyces avermitilis*, branched SCFAs were converted from BCAAs by the branched-chain alpha-keto acid dehydrogenase (BCKDH) complex (EC 1.2.4.4).^57^ However, the deletion of the BCKDH gene did not affect the production of branched SCFAs in *Clostridium sporogenes*.^22^ On the other hand, the *porA* gene, which is closely related to α-ketoisovalerate:ferredoxin oxidoreductase (VOR) (EC 1.2.7.7),^58^ was found to be essential. A mutation in the *porA* gene resulted in the cessation of 2-methylbutyrate, isobutyrate, and isovalerate production in *Clostridium sporogenes*. Additionally, a mutation in the *hadB* gene, which is involved in leucine fermentation, led to the loss of isocaproate production in this bacterium.^22^ Mutations in both genes in *Clostridium sporogenes* were shown to alter the host intestinal IgA response in a monocolonized gnotobiotic mouse model, suggesting a modulatory function of these branched SCFAs on host immunity.^22^ In a recent study, it was also found that the commensal *Parabacteroides merdae* produces branched SCFAs through the *porA* gene homolog.^59^ Additionally, the degradation of BCAA by this bacterium was shown to reduce atherosclerotic lesions in animal models^59^ (Table 1).

**Table 1.** The gut microbial biotransformation of lipid metabolites.

Bacterial name	Gene name	Precursor	End metabolite	Reference
*C. sporogenes*	*talA*	Threonine	Propionate	^22^
*C. sporogenes*	*croA*	3-Hydroxybutyryl-CoA	Butyrate	^22^
*B. thetaiotaomicron*	*mutA*/*scpA*	Succinyl-CoA	Propionate	^56^
*C. sporogenes*	*porA*	Leucine; Isoleucine; Valine	Isovalerate; 2-Me-butyrate; Isobutyrate	^22^
*C. sporogenes*	*hadB*	Leucine	Isocaproate	^22^
*P. merdae*	*porA*	Leucine; Isoleucine; Valine	Isovalerate; 2-Me-butyrate; Isobutyrate	^59^
*Lactobacillus*; *Bifidobacterium*; *Enterococcus*; *Propionibacterium*; *C. sporogenes*	*lai*	LA; LNA	CLAs; CLNAs	^26^
*L. plantarum*	*cla-hy*/*cla-dh*/ *cla-dc*/*cla-er*	LA	HYA; HYB; HYC; KetoA; KetoB; KetoC; CLAs	^60,61^
*Bacteroides*	*hm-NASs*	Glycine; FA acyl chain	*N*-3-hydroxypalmitoyl glycine	^28,29^
Unknown	*hm-NASs*	Alanine; FA acyl chain	*N*-myristoyl alanine	^28^
*Gemella*	*hm-NASs*	Serinol; FA acyl chain	*N*-palmitoyl serinol	^28^
*Neisseria*	*hm-NASs*	Ornithine; FA acyl chain	*N*-3-hydroxy palmitoyl ornithine	^28^
*Klebsiella*	*hm-NASs*	Glutamine; FA acyl chains	*N*-acyloxyacyl glutamine	^28^
*P. gingivalis*	unknown	Serine; Glycine; FA acyl chains	Lipid 654; Lipid 430; Lipid 1256	^27,62^
*Bacteroides*; *Lactobacillus*; *Clostridium*	*bsh* (hydrolase activity)	T/GCA; T/GCDCA; T/GUDCA; T/GDCA; T/GLCA; TβMCA	CA; CDCA; UDCA; DCA; LCA; βMCA	^34,52^
*R. gnavus*; *E. lenta*; *E. coli*; *Bacteroides*; *Clostridium*	*3*, *7*, or *12α*/*β-hsdh*	CA; CDCA	3-, 7-, or 12-Oxidated or epimerized BAs	^34^
*C. scindens*; *C*. *leptum*	*bai*	CA; CDCA	DCA; LCA	^32,34^
*R. gnavus*; *E. lenta*	*3α*/*β-hsdh*	DCA	3-OxoDCA; IsoDCA	^63,64^
*R. gnavus*; *E. lenta*	*3α*/*β-hsdh*	LCA	3-OxoLCA; IsoLCA	^65^
*Bacteroides*	*5β/α-reductase*; *3β-hsdh*	3-OxoLCA	IsoalloLCA	^66^
*C. perfringens*; *B. fragilis*	*bsh* (acyltransferase activity)	CA; CDCA; UDCA; DCA; Amines	Amine-conjugated BAs	^67,68^
*B. uniformis*	*BAS-suc*	CA; Succinic acid	3-Succinylated CA	^50^
Cluster IV *Clostridium*	*ismA*	Cholesterol	Coprostanol	^69,70^
*B. thetaiotaomicron*	*btSULT*	Cholesterol; IsoalloLCA; DHEA	Cholesterol-sulfate; IsoalloLCA-sulfate; DHEA-sulfate	^36,38^
*Bacteroides*; *Clostridium*	*gmGUS*	Estrogen glucuronides	Estrone; Estradiol	^71,72^
*B. fragilis*; *E. coli*; *H. hathewayi*; *C. massiliensis*; *P. niger*	*sulf*	Estrone-sulfate	Estrone	^73,74^
*M. neoaurum*	*3β-hsdh*	Testosterone	Androstenedione	^75^

### 3.2. Long-chain fatty acids

The human body’s essential fatty acids include linoleic acid (LA, C18:2 c9, c12) and linolenic acid (LNA, C18:3 c9, c12, c15), which are the precursors of omega-6 and omega-3 polyunsaturated fatty acids, respectively. After dietary intake, they can form dihydroxy gamma-linolenic acid (DGLA) and arachidonic acid (AA), or eicosapentaenoic acid (EPA) and docosahexaenoic acid (DHA) through FA synthesis pathways in the host.^76^ The host and microbes can further convert arachidonic acid to eicosanoid molecules such as prostaglandin derivatives.^31,77^ Symbiotic microorganisms also biotransform LA and LNA released from lipolysis, changing the position of their C=C unsaturated bonds in the fatty acid chain to form a series of microbially derived FA isomers.^26^ The LA isomers, also known as conjugated LAs (CLAs), include c9, t11 CLA, t10, c12 CLA, and t8, t10 CLA, while the LNA isomers, also known as conjugated LNAs (CLNAs), include c9, t11, c15 CLNA and c9, t13, c15 CLNA^26^ (Figure 1).

Microbial fatty acid isomerization is mainly mediated by linoleate isomerase (LAI) encoded by symbiotic microorganisms, which can convert LA and LNA to CLAs and CLNAs, respectively.^26^ Sequence alignment shows that LAI is highly homologous to myosin cross-reactive antigen (MCRA). Both have hydratase activity and are involved in the synthesis of hydroxylated fatty acids.^26^ In addition to LAI, research has shown that other proteins may also be involved in the fatty acid isomerization process, initiating a multi-step enzymatic reaction that ultimately changes the position of the C=C unsaturated bond to form fatty acid isomers.^60^ Symbiotic microorganisms with LAI include *Lactobacillus*, *Bifidobacterium*, *Enterococcus*, *Propionibacterium*, and a small number of *Clostridium* species such as *Clostridium sporogenes*, etc.^26,78^

The microbial conversion of LA to CLAs also produces a variety of hydroxylated or oxidized fatty acid intermediates. For instance, *Lactobacillus plantarum* has been shown to produce fatty acid intermediates, such as 10-hydroxy-cis-12-octadecenoic acid, 10-hydroxyoctadecanoic acid, and 10-oxooctadecanoic acid, during the conversion of LA to CLAs. This biotransformation was mediated by four enzymes, namely CLA-HY (LAI), CLA-DH, CLA-DC, and CLA-ER, in *Lactobacillus plantarum*. Studies have also shown that germ-free (GF) mice have lower levels of these hydroxy fatty acids in their guts.^60,61^ Research on the biological activities of these microbially derived long-chain fatty acid intermediates is emerging. Recent studies have shown that microbial LA metabolism can reduce the obesity-related inflammatory response caused by excessive LA intake.^79^ Mechanistically, 10-hydroxy-cis-12-octadecenoic acid (also known as HYA), a hydroxylated derivative of LA, activated the free fatty acid-sensing receptors GPR40 and GPR120 in the gut. The HYA-GPR40/GPR120 signaling then promoted the secretion of intestinal glucagon-like peptide-1 (GLP-1) to regulate host metabolic homeostasis.^79^ Similarly, another oxidized LA metabolite, 10-oxo-cis-12-octadecenoic acid (KetoA), was shown to increase energy expenditure in mice via transient receptor potential vanilloid 1 (TRPV1).^80^ A recent study also found that in addition to LA and LNA metabolites, other microbial-derived LCFA metabolites also have peroxisome proliferator-activated receptor (PPAR) agonist activity, suggesting that gut microbes, particularly lactic acid bacteria, may influence host physiology through a variety of LCFA metabolites.^81^

In addition to modifying the fatty acyl chain, bacteria can conjugate various amine groups to the carboxyl group of LCFAs to generate fatty amides via *N*-acyl synthase (NAS).^28^ Similar to host-derived fatty amines, these bacteria-derived fatty amines can also serve as GPCR agonists. For example, both host endogenous *N*-hexadecanoyl glycine and commensal bacteria-derived *N*-3-hydroxypalmitoyl glycine (as known as Commendamide) or *N*-myristoyl alanine activated GPR132/G2A, a GPCR with immunomodulatory functions.^28,29,82,83^ Bacterial *N*-palmitoyl serinol could activate GPR119, a GPCR involved in intestinal GLP-1 production, similar to host *N*-9-octadecenoyl ethanolamine.^28,84^ GF mice colonized with *N*-palmitoyl serinol-producing *E. coli* have been reported to have decreased levels of blood glucose due to increased GLP-1 secretion.^28^ Bacterial fatty amines also exhibit distinct agonist selectivity or even receptor antagonist properties compared to host ligands for the same type of GPCRs. For example, bacterial *N*-3-hydroxypalmitoyl ornithine specifically activated sphingosine-1-phosphate receptor (S1PR) 4, while the host ligand, S1P, activated all five members of the S1PR family.^28^ In addition, microbial *N*-acyloxyacyl glutamine has been shown to inhibit both prostaglandin I_2_ receptor (PTGIR) and prostaglandin E receptor 4 (PTGER4)^28^ (Table 1).

## 4. Immunomodulation of microbial SCFAs

### 4.1. Innate immunity

SCFAs have been shown to affect different cell types involved in innate immunity via several metabolite-sensing receptors such as GPR41, GPR43, and GPR109A or histone deacetylases (HDACs) inhibition.^54^ Intestinal epithelial cells (IECs) form a physical and biochemical defense barrier that separates host tissues from commensal microorganisms.^85^ SCFAs have been shown to promote intestinal epithelial integrity via the NLRP3 inflammasome activation and IL-18 release.^86^ The barrier-promoting effects of SCFAs require epithelial GPR43 and GPR109A signaling and downstream potassium ion efflux and ERK phosphorylation^86,87^ (Figure 1). Secretory IECs such as goblet cells and Paneth cells support barrier function by producing mucins and antimicrobial peptides. One recent study showed that the SCFA propionate promotes intestinal goblet cell differentiation and mucus-relevant gene expression via epithelial GPR41 signaling.^56^

Macrophages are classic innate immune cells that can engulf invading pathogens and senescent or damaged host cells, present antigens to T cells, and mount proper immune responses to maintain tissue homeostasis. Macrophages express a myriad of pattern recognition receptors (PRRs) to recognize pathogen or damage-associated molecular patterns (PAMPs or DAMPs). In response to different stimuli present in the microenvironment, macrophages can be polarized into inflammatory macrophages (M1) or alternatively activated macrophages (M2). Several reports support the anti-inflammatory role of SCFAs in modulating macrophage activity in both *in vitro* and *in vivo* models. A study by Pamela *et al*. showed that butyrate suppresses LPS-induced IL-6, iNOS, and IL-12, but not TNF-α or MCP-1 production by colonic CX_3_CR1^hi^CD103^−^ macrophages.^88^ This effect was independent of either TLR4 or GPR109A signaling but was due to the inhibition of HDACs by butyrate.^88^ However, another study found that butyrate-induced expression of IL-10 and ALDH1A1 requires GPR109A signaling in intestinal macrophages and dendritic cells (DCs).^89^ These observations suggest that the anti-inflammatory function of butyrate in macrophages may be gene and cell-type context-dependent. In addition to resolving inflammation, SCFAs have been reported to play beneficial roles in antibacterial and antiviral immunity. Schulthess *et al*. found that macrophages differentiated *in vitro* in the presence of butyrate exhibit enhanced antimicrobial activity without increased inflammatory responses.^90^ Butyrate imprinted the antimicrobial state through HDAC3 inhibition, driving metabolic switch and LC3-associated phagocytosis in macrophages for enhanced microbicidal function against *Salmonella* infection *in vivo*.^90^ Butyrate-induced HDAC3 inhibition in macrophages has also been implicated in an antimicrobial program to control *Staphylococcus aureus*-induced mastitis.^91^ Microbiota-derived acetate has been reported to induce antiviral responses via GPR43-mediated type I interferon production.^92,93^

Innate lymphoid cells, including ILC1, ILC2, and ILC3, are characterized by the expression of signature transcriptional factors and cytokines similar to their T helper cell counterparts. ILCs are primarily found in mucosal tissues such as the gut, lung, and skin and are therefore putative responders to commensal-derived metabolites. Two groups reported that ILCs, particularly ILC2 and ILC3, express the SCFA receptor GPR43 at high levels.^94,95^ Activation of GPR43 by SCFAs or the chemical agonist selectively promoted ILC3 expansion and IL-22 expression. Mice deficient in *Gpr43* had less IL-22^+^ ILC3 and were consequently more susceptible to dextran sulfate sodium (DSS)-induced colitis and *Citrobacter rodentium* infection.^94^ Mechanistically, SCFAs triggered the expression of IL-22 in ILC3 via AKT and STAT3 signaling.^94^ Similarly, acetate was reported to coordinate ILC3 responses against *Clostridioides difficile* infection by promoting the expression of IL-1 R in ILC3, which further facilitated IL-22 production in response to IL-1β stimulation.^96^ However, in the context of the ILC2 scenario, butyrate was reported to inhibit HDCA activity, thus downregulating the expression of type 2 cytokines IL-5 and IL-13 in ILC2 to alleviate allergic inflammation.^97^

### 4.2. Adaptive immunity

The immunomodulatory role of SCFAs in adaptive immunity has also been demonstrated (Figure 1). Effector CD4^+^ T cells are critical for host defense and the development of autoimmune diseases, while FOXP3^+^ regulatory T cells (Tregs) are immunosuppressive T cells that maintain immune homeostasis. GF mice, which lack all commensal microorganisms, were reported to be defective in colonic Treg development, whereas microbiota colonization restored Treg levels.^98,99^ Supplementation of SCFAs in drinking water normalized Treg numbers in GF mice and increased IL-10 expression in Tregs. This effect was GPR43 dependent, as SCFAs failed to restore Treg numbers in *Gpr43*-deficient mice.^100^ Yukihiro *et al*. also found that butyrate induces the differentiation of colonic Tregs by upregulating histone H3 acetylation levels at both the promoter and conserved non-coding sequence regions (CNS1 and CNS3) of the *Foxp3* gene locus.^101^ In addition, a third study showed that butyrate and propionate could increase extrathymic CNS1-dependent differentiation of Tregs via their HDAC inhibitory activity.^102^ Recently, pentanoate, also known as valerate, a less characterized SCFA, was also shown to promote c-Maf-expressing colonic Treg differentiation via increasing iron uptake.^103^ Besides Tregs, SCFAs were also found to regulate the functions of effector CD4^+^ T cells. In a naïve T cell differentiation system, SCFAs could promote Th1 and Th17 differentiation. Interestingly, these SCFA-differentiated effector T cells were also mixed with IL-10-producing CD4^+^ T cells. Similarly, SCFAs inhibited HDAC activity to enhance mTOR-S6K signaling to increase the expression of the effector T cell cytokines.^104^ A higher concentration of butyrate induced Th1-like IFN-γ production in either Treg or conventional T effector cells. This effect was mediated by HDAC inhibition of butyrate and H3 acetylation at the *Tbx21* and *Ifng* locus and was independent of the SCFA receptors GPR41 and GPR43.^105^ Later, two studies found that butyrate signals through GPR43 to induce IL-10 production in Th1 cells or through GPR41 and HDAC inhibition to promote IL-22 production in CD4^+^ T cells, requiring downstream activation of STAT3 and mTOR in these cells.^106,107^

CD8^+^ T cells are important for the control of intracellular pathogens and tumor growth. Serum acetate levels were found to increase rapidly after bacterial infection. Upon uptake by memory CD8^+^ T cells, acetate increased the acetyl-coenzyme A (acetyl-CoA) pool and promoted the acetylation of GAPDH and glycolysis rate, which further facilitated the rapid memory CD8^+^ T response and IFN-γ production to control *Listeria monocytogenes* infection.^108^ The transition of activated CD8^+^ T cells into memory cells has also been reported to be microbiota-dependent. High-fiber diet (HFD)-induced butyrate could promote the memory potential of activated CD8^+^ T cells via GPR41 and GPR43. Mechanistically, butyrate mediated metabolic rewiring of activated CD8^+^ T cells and facilitated sustained glutamine utilization and fatty acid oxidation.^109^ HFD-derived butyrate has been shown to enhance the CD8^+^ T cell response to influenza infection via the GPR41 receptor and altered cellular metabolism.^110^ SCFAs could also directly induce IFN-γ expression in CD8^+^ cytotoxic T cells (CTLs) and CD8^+^IL-17^+^ T cells (Tc17) by inhibiting HDAC activity, similar to their effect on CD4^+^ T cells.^111^ Recent studies suggest a strong link between the microbiome and antitumor therapy. It was shown that antibiotic-treated mice were less responsive to anticancer chemotherapy, and butyrate supplementation restored their response to chemotherapy.^112^ Butyrate upregulated the ID2-IL-12R cascade through the epigenetic remodeling of CD8^+^ T cells, which further promoted the functionality of CD8^+^ T cells to control tumor growth.^112^ In addition, a similar role of pentanoate and butyrate in enhancing the anti-tumor activity of CTLs and chimeric antigen receptor (CAR) T cells has been reported.^113^ By activating mTOR kinase and inhibiting HDAC activity, pentanoate and butyrate induced metabolic and epigenetic reprogramming of CTLs and CAR T cells to enhance TNF-α and IFN-γ production.^113^ However, there are conflicting observations of SCFAs in anti-tumor immunity. Systemic administration of SCFAs was reported to limit the anti-tumor effect of CTLA-4 blockade, which was associated with increased Treg frequency in the periphery. In addition, butyrate also inhibited CD80/CD86 expression on DCs, which impeded T cell priming and activation.^114^ The discrepancy in the conflicting results may be due to differences in experimental settings, such as anti-tumor therapy approaches, mouse tumor models, or SCFA doses/types used in each study. These parameters not only directly affect CD8^+^ T cell responses, but also likely affect other cell types that inhibit tumor killing, and thus in combination may create different *in vivo* biological contexts during antitumor therapy, leading to discrepant cancer therapy outcomes.

B cells can produce immunoglobulins (Ig) against pathogens and commensals to maintain intestinal homeostasis. SCFAs could directly increase acetyl-CoA levels and promote overall metabolic fitness including glycolysis, oxidative phosphorylation, and fatty acid synthesis to fuel plasma cell differentiation and IgA and IgG production. In addition to metabolic regulation, SCFAs also inhibited HDACs to upregulate the expression of *Prdm1*, *Aicda*, and *Xbp1*, which were positively associated with B cell differentiation.^115^ SCFAs may also indirectly promote antibody production by regulating DC function. Acetate was shown to increase IgA-producing B cells in the intestine of wild-type but not *Gpr43*-knockout mice. They further suggested that acetate increases the expression of *Aldh1a2* in DCs, a key enzyme that converts vitamin A to retinoid acid, and promotes IgA production by B cells.^116^ However, a recent study showed that the SCFAs butyrate and propionate differentially regulate the antibody response in a dose-dependent manner. While low doses of SCFAs enhanced class-switch DNA recombination (CSR), high doses of SCFAs impaired *Aicda* and *Prdm1* expression and CSR, leading to reduced antibody production. High-dose SCFAs acted through epigenetic upregulation of miRNAs that target *Aicda* and *Prdm1* mRNA for degradation, thereby dampening B cell functions^117^ (Table 2).

**Table 2.** The immunomodulatory functions of microbial lipid metabolites.

Metabolite	Innate/adaptive	Cell type	Mechanism	Reference
SCFAs	Innate	IEC	Signal through GPR43 and GPR109A to activate NLRP3	^86,87^
Propionate	Innate	IEC	Signals through GPR41 to promote goblet cell differentiation	^56^
Butyrate	Innate	Macrophage	Inhibits HDACs to suppress inflammation	^88^
Butyrate	Innate	Macrophage; DC	Signals through GPR109A to suppress inflammation	^89^
Butyrate	Innate	Macrophage	Inhibits HDAC3 against bacterial infection	^90,91^
Acetate	Innate	Pulmonary EC; Macrophage	Signals through GPR43 against viral infection	^92,93^
SCFAs	Innate	ILC3	Signal through GPR43 to promote IL-22^+^ ILC3	^94,96^
Butyrate	Innate	ILC2	Inhibits HDACs to suppress ILC2	^97^
SCFAs	Adaptive	Treg	Signal through GPR43 to promote Treg differentiation	^100^
Butyrate	Adaptive	Treg	Inhibits HDACs to promote Treg differentiation	^101,102^
Pentanoate	Adaptive	Treg	Enhances iron uptake to promote Treg differentiation	^103^
SCFAs	Adaptive	Th1; Th17	Inhibit HDACs to promote CD4^+^ effector T differentiation	^104,105^
SCFAs	Adaptive	IL-10^+^ Th1	Signal through GPR43 to induce IL-10^+^ Th1	^106^
SCFAs	Adaptive	IL-22^+^ CD4^+^ T	Signal through GPR41 and HDAC inhibition to induce IL-22^+^ CD4^+^ T	^107^
Acetate	Adaptive	CD8^+^ T	Promotes glycolysis	^108^
Butyrate	Adaptive	CD8^+^ T	Signals through GPR41 and GPR43 to promote memory CD8^+^ T	^109^
Butyrate	Adaptive	CD8^+^ T	Signals through GPR41 to enhance CD8^+^ T mediated anti-viral responses	^110^
Butyrate	Adaptive	CD8^+^ T	Inhibits HDACs to promote IFN-γ and IL-17 production in CD8^+^ T	^111^
Butyrate	Adaptive	CD8^+^ T	Inhibits HDACs to upregulate ID2 and IL-12 signaling in CD8^+^ T	^112^
Butyrate; Pentanoate	Adaptive	CD8^+^ T; CAR T	Inhibit HDACs to promote TNF-α and IFN-γ production in CD8^+^ T and CAR T	^113^
Butyrate; Pentanoate	Innate; Adaptive	DC; CD4^+^ T; CD8^+^ T	Inhibit CD80/CD86 in DCs and impede T cell activation during immune therapy	^114^
SCFAs	Adaptive	B cell	Inhibit HDACs to promote B cell differentiation	^115^
Acetate	Innate; Adaptive	DC; B cell	Signals through GPR43 to enhance IgA responses	^116^
Butyrate; Propionate	Adaptive	B cell	Modulate B cell functions in a dose-dependent manner	^117^
c9, t11-CLA	Innate	DC	Inhibits NF-κB activation	^118^
HYA	Innate	IEC	Signals through the GPR40-ERK pathway	^119,120^
13-Hydroxy-9(*Z*),15(*Z*)-octadecadienoic acid; 13-Oxo-9(*Z*),15(*Z*)-octadecadienoic acid	Innate	Macrophage	Signal through GPR40 to promote M2 macrophage differentiation	^121^
γHYD; γKetoD	Innate	IEC	Signal through PPARδ to enhance IEC FA β-oxidation	^81^
Lipid 430; Lipid 654; Lipid 1256	Innate	TLR2-HEK cell; Monocyte	Signal through TLR2 to induce inflammation	^27,62^
c9, t11-CLA; t10, c12-CLA	Adaptive	CD4^+^CD8αα^+^ T	Signal through HNF4G to promote CD4^+^CD8αα^+^ IEL differentiation	^78^
Unconjugated BAs	Innate	IEC	Increase gut permeability	^122^
DCA	Innate	IEC	Signals through FXR to induce Paneth cell dysfunction	^123^
6-Ethyl-CDCA	Innate	Macrophage	Signals through FXR to suppress inflammation	^124^
TLCA; LCA; DCA	Innate	Macrophage	Signal through TGR5 to suppress inflammation	^125^
LCA	Innate	Macrophage	Signals through TGR5 to suppress NLRP3	^126^
DCA	Innate	DC	Signals through TGR5 to suppress NF-κB	^127^
DCA; CDCA	Innate	Macrophage	Activate NLRP3 by promoting calcium influx	^128^
CA	Innate	ILC2	Signals through FXR to activate ILC2	^129^
3-OxoLCA; IsoLCA	Adaptive	Th17	Inhibit RORγt	^65,130^
IsoalloLCA	Adaptive	Treg	mtROS and NR4A1-dependent *Foxp3* CNS3 enhancement	^66,130^
CA; CDCA; UDCA	Adaptive	Treg	Signal through VDR to stabilize the RORγt-program in Tregs	^131^
IsoDCA	Innate; Adaptive	DC; Treg	Signals through FXR to enhance DC-instructed Treg differentiation	^63^
BAs	Adaptive	CD4^+^ T	CAR-directed T cell adaptation to BAs	^132^
Trp-CDCA	Adaptive	CD4^+^ T	Likely signals through PXR to induce IFN-γ^+^ Th17 cells	^133^
LCA; DCA	Innate; Adaptive	CRC cell; Treg	Signal through TGR5 to induce CCL28 for intra-tumoral Treg recruitment	^134^
DCA	Adaptive	CD8^+^ T	Modulates calcium-NFAT signaling to suppress CD8^+^ T functions	^135^

## 5. Immunomodulation of microbial LCFAs

### 5.1. Innate immunity

LCFAs, such as LA and LNA, are essential polyunsaturated fatty acids (PUFAs) that people get from their diets. They may serve as precursors of omega-6 and omega-3 PUFAs, respectively. Both types of lipid mediators could modulate inflammation in a context-dependent manner. CLAs or CLNAs are microbial isomers of LA or LNA with anti-inflammatory properties that may benefit health.^26,136^ *In vitro* studies suggested that CLAs could modulate inflammatory cytokine production in myeloid cells. The cis-9, trans-11 isomer of CLAs (c9, t11-CLA) could inhibit IL-12 production in LPS-treated bone marrow-derived DCs. The c9, t11-CLA was shown to suppress NF-κB activation and promote IL-10 production through the ERK pathway.^118^ HYA, a hydroxylated metabolite of LA, was found to improve epithelial barrier function via the GPR40-ERK pathway,^119,120^ while LNA derivatives such as 13-hydroxy-9(Z),15(Z)-octadecadienoic acid and 13-oxo-9(Z),15(Z)-octadecadienoic acid were also shown to induce anti-inflammatory M2 macrophage differentiation via the same signaling pathway.^121^ Both CLAs and CLNAs, as well as these intermediate derivatives, have been reported to be potential agonists for PPARs,^81^ likely protecting mice from DSS-induced colitis via activation of the PPAR-mediated pathways in macrophages.^137^

In addition to their GPCR modulatory activities, microbial fatty amides can also activate host innate receptors. *Porphyromonas gingivalis* (*P. gingivalis*) is a periodontopathic pathogen involved in chronic periodontal disease.^138^ *P. gingivalis*-derived fatty amides such as *N*-3-hydroxy-15-methylhexadecanoyl glycylserine (lipid 430) and *N*-15-methyl-3-((13-methyltetradecanoyl)oxy)hexadecanoyl glycylserine (lipid 654) contributed to periodontal inflammatory responses via activating host TLR2.^27^ Another fatty amide lipid produced by *P. gingivalis*, phosphoglycerol serine-glycine lipodipeptide (lipid 1256), was a 50-fold more potent agonist in engaging TLR2 than lipid 654.^62^

### 5.2. Adaptive immunity

The role of microbial LCFAs in modulating host adaptive immunity remains largely unexplored. We recently found that the gut microbial fatty acid isomerization pathway and its end products, CLAs, modulate the development of a subset of intraepithelial lymphocytes (IELs), termed CD4^+^CD8αα^+^ IELs, in the small intestine^78^ (Figure 1). Previous studies and our data suggest that the development of CD4^+^CD8αα^+^ IELs is dependent on microbial and dietary cues,^139–141^ supported by the fact that GF or SPF mice fed a chemically simplified diet had impaired CD4^+^CD8αα^+^ IEL development. In the monocolonized gnotobiotic mouse model, only symbionts with intact LCFA isomerization pathways could restore CD4^+^CD8αα^+^ IEL numbers, suggesting that isomerization of LCFAs by microbes is required for the induction of CD4^+^CD8αα^+^ IELs. In addition, supplementation of CLAs in drinking water also normalized CD4^+^CD8αα^+^ IELs in mice fed a chemically simplified diet.^78^ Mechanistically, we found that intestinal CD4^+^CD8αα^+^ IELs selectively express the transcription factor HNF4G, which can bind to CLAs and promote intrinsic *Il18r1* transcription. While IL-18 signaling downregulated the CD4 lineage committed transcription factor ThPOK, thereby supporting CD4^+^CD8αα^+^ IEL development. Consequently, mice deficient in *Hnf4g* or *Il18r1* had impaired CD4^+^CD8αα^+^ IEL development and were susceptible to *Salmonella typhimurium* infection.^78^ The transcription factor HNF4A, analog to HNF4G, on the other hand, has been reported to promote the differentiation of natural IELs, namely TCRβ^+^CD8αα^+^ and TCRγδ^+^ cells, by regulating the expression of butyrophilin-like molecules in intestinal epithelial cells.^142^ In addition to CLAs, the immunological functions of other microbial LCFAs, especially various intermediate metabolites, and their host sensing mechanisms in modulating host adaptive immunity need further investigation (Table 2).

## 6. Microbial transformations of gut steroids

### 6.1. Bile acids

Bile acids are sterol molecules formed from cholesterol through a cascade of enzymatic reactions in the liver. The two major primary bile acids synthesized by the human liver are cholic acid (CA) and chenodeoxycholic acid (CDCA). At the same time, these primary bile acids undergo a further conjugation reaction with glycine or taurine to form conjugated primary bile acids such as TCA, TCDCA, GCA, and GCDCA, etc., which are secreted from the liver into the gallbladder for storage.^34,143^ The gallbladder contracts in response to food intake, releasing bile acids into the small intestine to help the host absorb dietary fatty acids and fat-soluble vitamins. 95% of bile acids in the small intestine are reabsorbed by the liver via the enterohepatic circulation, while approximately 5% of bile acids enter the colon and are biotransformed by commensal microorganisms to microbial bile acid metabolites.^34,143^

The bile acid biotransformation pathways of symbiotic microorganisms include three main categories: 1) The bile acid deconjugation reaction mediated by bile salt hydrolase (BSH) converts the conjugated primary bile acids synthesized in the liver to deconjugated primary bile acids. Microbial BSHs belong to the choloylglycine hydrolase family (EC3.5.1.24) and can hydrolyze the C-24 *N*-acyl amide bond that links primary bile acids to their taurine and/or glycine conjugates. 2) Oxidation and epimerization of the 3-, 7-, or 12-hydroxyl group of bile acid molecules mediated by 3α/β-, 7α/β-, or 12α/β-hydroxysteroid dehydrogenases (HSDHs) to form various dehydrogenated or hydroxyl isomerized bile acid molecules. The conversion of bile acid hydroxy groups from α to β configuration involves a stable oxidized bile acid intermediate. These stereochemistry changes, mediated by microbial HSDHs, are also reversible from β to α configuration. The epimerization reaction can occur within a single bacterium that possesses both α and β-HSDHs or can be achieved through the coexistence of different bacteria, where one harbors α-HSDHs while the other has β-HSDHs. 3) The 7α-dehydroxylation pathway removes the 7α-hydroxy or 7β-hydroxy group of primary bile acids to form secondary bile acids, including deoxycholic acid (DCA) and lithocholic acid (LCA). The process requires a bile acid-inducible operon called *Bai*, which consists of eight genes.^34,143–145^ Recently, researchers have discovered that intestinal microorganisms can also mediate novel bile acid conjugation reactions that are independent of glycine or taurine, forming phenylalanocholic acid, tyrosocholic acid, or leucocholic acid, and other novel conjugated BAs.^33,133,146,147^ Subsequent studies have shown that microbial BSHs not only have hydrolase activity, but can also serve as *N*-acyltransferases that conjugate amines to BAs, forming microbial conjugated BAs.^67,68^ In addition to amine-conjugated BAs, microbial acylated BAs such as 3-acetylated CA and 3-succinylated CA were also discovered by a click chemistry-based mass spectrometry approach.^50^ The biosynthesis of 3-succinylated CA was mediated by the BA acyl synthetase for succinyl (BAS-suc) in *Bacteroides uniformis*.^50^

**Figure 2.**
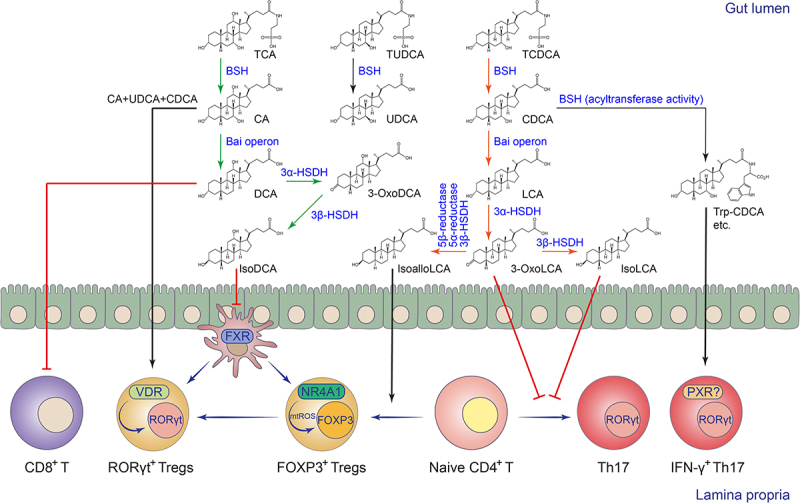
Biotransformation and immunomodulation of microbial BAs in the intestine.

The enzymatic diversity and specificity of microbial bile acid metabolism determine the relative abundance of different bile acid metabolites and shape the intestinal bile acid landscape.^52,144,148^ Compared to the microbial 7α-dehydroxylation pathway for secondary bile acid production, the microbial pathways for bile acid deconjugation, oxidation, and epimerization are widely distributed in various intestinal commensal bacteria, including *Bacteroides*, *Clostridium*, *Lactobacillus*, *Bifidobacterium*, and others.^34,144^ The microbial secondary bile acid production pathway exists mainly in a small number of intestinal commensal *Clostridium* species such as *Clostridium scindens* and *Clostridium leptum*.^34^ The biotransformation cascade is mediated by the 7α-dehydroxylation pathway, encoded by the *Bai* operon in *Clostridium*, which converts CA and CDCA to DCA and LCA, respectively. The enzymatic cascade and its bile acid intermediates of the 7α-dehydroxylation pathway have recently been elucidated.^32^ The secondary bile acids DCA and LCA, in turn, undergo 3α/β-HSDH-mediated oxidation and epimerization to yield various bile acid derivatives, such as isoDCA, isoLCA, 3-oxoLCA, and isoalloLCA, which have recently been shown to have immunomodulatory functions in the gut^63,65,66^ (Figure 2 and Table 1).

Host-derived conjugated BAs, such as TCA, TUCDA, and TCDCA, are deconjugated by microbial BSH to produce CA, UDCA, and CDCA, while CA and CDCA are further converted to the secondary BAs DCA and LCA, respectively. DCA can inhibit the effector function of CD8^+^ T cells, while also undergoing oxidation and epimerization to generate isoDCA, which has gut Treg-promoting functions. LCA is converted to 3-oxoLCA and isoLCA to suppress gut Th17 differentiation, or isoalloLCA to promote gut Treg differentiation via mtROS and NR4A1. The BA pool also contributes to the development of RORγt^+^ Tregs via VDR. CDCA and other gut BAs can be microbially conjugated with amino acids such as tryptophan, etc. The resulting metabolites, such as Trp-CDCA, promote IFN-γ production in Th17 cells *in vitro*.

### 6.2. Cholesterol and hormones

Cholesterol is the chemical precursor for the synthesis of bile acids and other steroid molecules. Circulatory cholesterol levels are an important indicator of human health. Intestinal microorganisms have been shown to regulate serum cholesterol levels through microbial cholesterol metabolism. For example, recent metabolomics and bioinformatics studies found that certain uncultured members of Cluster IV *Clostridium* within our gut microbiota carry genes for cholesterol dehydrogenases, namely intestinal sterol metabolism A (*ismA*), which can convert cholesterol to coprostanol.^69,70^ Individuals harboring *ismA*-encoding bacteria had significantly lower fecal and serum cholesterol levels.^69,70^ In addition to cholesterol dehydrogenation, other gut bacteria such as *Bacteroides* have been shown to convert cholesterol to cholesterol-3-sulfate via their sulfotransferases (SULTs).^36,38^ The cholesterol sulfonation gene cluster in *Bacteroides* also sulfonated other steroid molecules such as isoalloLCA, etc.^38^ Since LCA derivatives have been reported to be associated with healthy aging in centenarians,^149^ and, as discussed below, with immunomodulatory activity, it is intriguing to investigate whether bacterial sulfonation alters their bioactivity and impacts our health.

Estrogens and androgens are important for host homeostasis and development. Gut microbes can metabolize them to various derivatives and thus affect systemic levels of sex steroid hormones. Under normal circumstances, estrogens are rapidly deactivated in the liver and eliminated from the body through urine and feces. Estrogens are inactivated by glycosidation, sulfation, or methylation of their sterol core structure.^150^ However, gut microbes can remove the glucuronides or sulfates from steroids to reactivate estrogens via their deconjugation enzymes. The removal of glucuronides from deactivated estrogens was mediated by microbial β-glucuronidase (GUS) enzymes, which are abundant in human gut microbiota members such as *Clostridium* and *Bacteroides*.^72,151^ One study has reported heterologous expression of 35 gut microbiota-derived GUS enzymes in *E. coli*, nearly half of which could reactivate estrone or estradiol by removal of glucuronides.^71^ Gut microbes have also been shown to deconjugate sulfated estrogens or sulfonate estrogen-related molecules via opposite enzymatic reactions. For example, *Bacteroides fragilis* or *Peptococcus niger* encoded sulfatases (SULF) that convert estrone sulfate to estrone,^73,74^ while *Bacteroides thetaiotaomicron* sulfonated estrogen precursor dehydroepiandrosterone (DHEA) via its sulfotransferase.^38^ Similar to estrogens, androgens could be inactivated by conjugation, while gut bacteria could degrade them to pyruvate and propionyl-CoA for energy production via the super pathway of testosterone and androsterone degradation.^152,153^ In addition, *Mycobacterium neoaurum*-derived 3β-HSDH has been reported to convert testosterone to androstenedione, causing a depression-like phenotype in rats.^75^ Endogenous hormone levels are closely associated with certain types of metabolic syndromes and cancers, while the impact of microbe-mediated hormone metabolism on the host immune system requires further investigation (Table 1).

## 7. Immunomodulation of microbial BAs

### 7.1. Innate immunity

The gut microbiota-derived BA metabolites have been reported to regulate several types of innate immune cells through either nuclear or membrane receptors. In general, intestinal BAs at physiological concentrations can promote IEC proliferation and epithelial integrity.^154^ Although exceeding levels of microbial-deconjugated BAs may increase epithelial permeability, host-conjugated BAs sequester these microbial BAs to form micelles, and thus maintain gut integrity.^122^ However, consumption of a Western diet (WD) led to overproduction of the secondary bile acid DCA, resulting in host intestinal Paneth cell dysfunction. Mechanistically, WD consumption increased the abundance of the *BaiCD* operon in the gut microbiome, and the resulting higher level of DCA promoted intestinal type I IFN response and Paneth cell defects in an FXR-dependent manner.^123^ Microbial bile acid-mediated signaling were also reported to negatively regulate the innate immune response in macrophages or DCs. For example, the BA receptor FXR has been shown to be directly involved in the negative regulation of NF-κB and AP-1 target genes.^124^ Activated FXR entered the nucleus and controlled the LPS-induced inflammatory response by stabilizing the inhibitory effect of the NCoR complex on the promoter region of inflammation-related genes.^124^ In addition to FXR, microbial BAs have been reported to inhibit LPS-induced inflammatory factor expression through cAMP signaling mediated by the membrane receptor TGR5.^125^ Mechanistically, a recent study found that the secondary BA LCA activates TGR5-cAMP-PKA signaling to phosphorylate serine at position 291 of the NLRP3 protein, leading to ubiquitination of NLRP3 and inhibition of inflammasome activation in bone marrow-derived macrophages.^126^ Another study reported that the secondary BA DCA activates the same pathway, leading to the inhibition of NF-κB activation in human monocyte-derived DCs.^127^ However, DCA has also been reported to activate the NLRP3 inflammasome by increasing calcium influx in THP-1-derived macrophages.^128^ The contrary observations suggest the context-dependent biological function of secondary bile acids. Nevertheless, the BA receptor FXR was shown to inhibit the NLRP3 inflammasome via physical interaction with NLRP3 and CASP1 in the study.^128^ Although dietary fiber may have beneficial anti-inflammatory effects via microbially fermented SCFAs, a recent study showed that inulin fiber alters the structure of the gut microbiota in mice, leading to an increase of a deconjugated primary BA, CA, which induces FXR-dependent IL-33 production, ILC2 activation and type 2 inflammation^129^ (Table 2).

### 7.2. Adaptive immunity

Emerging evidence also suggests that microbial bile acid metabolites are important regulators of the differentiation and function of adaptive immune cells, particularly CD4^+^ T cells, via receptor-dependent or independent mechanisms (Figure 2). The intestinal CD4^+^ T cell subsets, such as Th17 and Treg cells, are critical for intestinal anti-infection immunity and maintenance of intestinal immune homeostasis.^2^ Two BA derivatives of LCA, 3-oxoLCA, and isoalloLCA, have been reported to regulate the differentiation and development of intestinal Th17 and Treg cells, respectively.^130^ 3-OxoLCA can inhibit the differentiation and development of Th17 cells by directly binding to RORγt, a master transcription factor that controls Th17 cell fate.^130^ In addition, some human symbiotic microorganisms from the Actinobacteria or Clostridia phyla, such as *Eggerthella lenta* and *Ruminococcus gnavus*, have been shown to convert LCA to 3-oxoLCA and isoLCA through their 3α/β-HSDHs, and isoLCA had similar regulatory effects in inhibiting Th17 cell differentiation.^65^ IsoalloLCA, on the other hand, was shown to promote mitochondrial ROS (mtROS) generation in a BA receptor-independent manner, thereby enhancing the expression of the Treg cell signature transcription factor FOXP3 to promote Treg cell differentiation and development.^130^ Mechanistically, the conserved non-coding sequence 3 (CNS3) in the enhancer region of the *Foxp3* gene and the nuclear receptor NR4A1 have been reported to be involved in the isoalloLCA-induced Treg differentiation process.^66,130^ The study also showed that intestinal *Bacteroides* species can convert 3-oxoLCA to isoalloLCA via a multistep enzymatic reaction involving 5β-reductase, 5α-reductase, and 3β-HSDH.^66^ Deconjugation of host-derived bile acids by microbial BSH is the gateway reaction for microbial BA metabolism. The metabolic activities of different BSHs ultimately determine the size and composition of the intestinal bile acid pool.^34^ Our study revealed that the BSH pathway of *Bacteroides* is critical for maintaining the levels of colonic RORγt^+^ Treg cells, which have been shown to be mainly regulated by microbial colonization and play an important immunoregulatory function in the gut.^131,155,156^ We found that microbial-derived bile acid metabolites maintain the levels of RORγt^+^ Treg cells in the gut mainly through the BA receptor VDR.^131^ Intrinsic *Vdr*-deficiency dampened the RORγt-mediated transcriptional program in Treg cells, and predisposed mice to show exacerbated disease phenotypes in the DSS-induced colitis model.^131^ Microbial BAs may not only directly control the fate of Treg cells in the gut, but also indirectly modulate their differentiation, development, or recruitment. For example, the 3α/β-HSDHs of *Eggerthella lenta* and *Ruminococcus gnavus* were reported to convert DCA to isoDCA.^64^ IsoDCA, in turn, promoted the differentiation and development of peripheral Treg cells by inhibiting FXR-mediated gene expression in intestinal DCs.^63^ More recently, several newly identified amine-conjugated BAs have been shown to activate the nuclear receptor PXR, and among them, Trp-CDCA in particular, has been shown to induce IFN-γ in RORγt^+^CD4^+^ effector T cells and is likely to contribute to the pathogenesis of Inflammatory bowel disease (IBD).^133^ However, to minimize the cytotoxicity of high concentrations of bile acids in the intestine, the nuclear receptor CAR was activated in intestinal CD4^+^ effector T cells. This activation induced bile acid tolerance via the xenobiotic transporter MDR1 and controlled excessive intestinal inflammation via the immunosuppressive cytokine IL-10.^132^ During the progression of colorectal cancer (CRC), the presence of *Bacteroides*-derived BSH elevated unconjugated DCA and LCA levels. This, in turn, resulted in TGR5-dependent CCL28 production in colon tumors. The chemokine CCL28 recruited intra-tumoral Tregs to dampen CD8^+^ T cell-mediated anti-tumor immunity.^134^ Microbial bile acids can also directly modulate the activity of CD8^+^ T cells. A recent study demonstrated that DCA inhibits calcium-nuclear factor of activated T cells (NFAT)2 signaling by targeting plasma membrane Ca^2+^ ATPase (PMCA) in CD8^+^ T cells. As a result, microbial DCA suppressed CD8^+^ T cell effector function and promoted CRC development in mouse models^135^ (Table 2).

These studies revealed that several microbial BA biotransformation pathways, including the BA deconjugation pathway, the 7α-dehydroxylation pathway that produces secondary BAs, and the hydroxyl oxidation and epimerization pathways for these secondary BAs, are all involved in the production of BA metabolites with immunomodulatory activity. Whether strategies targeting relevant bile acid metabolic enzymes or altering bile acid flux can be used to effectively intervene in intestinal inflammatory diseases remains to be investigated.

## 8. Concluding remarks

In this review, we discuss recent research progress on immune modulation of intestinal barrier function by fatty acid and bile acid metabolites derived from gut microorganisms. Both types of microbial metabolites have diverse structures and are involved in the regulation of host innate and adaptive immunity. The diversity and complexity of lipid molecules produced by the symbiotic microbes of the human gut have presented both great opportunities and challenges to our research community. In recent years, the development of high-throughput microbial sequencing technology, such as 16S ribosomal RNA and metagenomic sequencing, and metabolomics technology have allowed us to identify the structural composition of a given microbial community and its associated lipid metabolites. Furthermore, we can correlate such microbial signatures with host mucosal immune phenotypes using bioinformatics technology. However, it is still difficult to efficiently explore the causal relationship between the diverse microbial lipid metabolites and their corresponding immunomodulatory functions/mechanisms using omics technologies alone. Currently, we face several technical obstacles in studying the host modulatory functions of these microbial lipid molecules. For example, the host’s intestinal commensal microorganisms are very diverse, and some of the species still cannot be isolated and cultured *in vitro*, making it difficult to evaluate their lipid metabolism capabilities. Second, although microbial genetic manipulation technologies for commensal bacteria are developing, the genomes of a large number of symbiotic microorganisms cannot be precisely edited, limiting our understanding of how commensal bacteria metabolize different lipid molecules and the host modulatory functions of such microbial transformation pathways. Finally, due to the diversity of microbial lipid metabolites, it is still challenging to annotate the chemical structures of a large number of *de novo* microbial lipid metabolites using untargeted mass spectrometry (MS) technology, making it difficult to establish a relationship between an uncharacterized microbial lipid molecule and its host receptor or target molecule.

Although these research challenges limit our comprehensive understanding of how commensal microbial-derived lipid molecules interact with the host immune system at the cellular and molecular levels, we should also see ample opportunities for exploring new methods to isolate unculturable symbiotic microorganisms, developing microbial genetic tools to edit symbiotic microorganisms, and identifying novel bioactive microbial lipids. Despite the knowledge gained from microbial sequencing data, further experimental validation and functional studies require new isolation and cultivation methods for previously uncultured microorganisms.^157^ Emerging microbial culturomics technologies, which integrate machine learning with automated platforms, have begun to fascinate the high-throughput cultivation of uncultured gut microbes.^158^ Using this culturomics framework, the researchers obtained 26,997 microbial isolates, representing over 80% of all abundant taxa, from the fecal samples of 20 human participants.^158^ This high-throughput microbial isolation platform, combined with high-resolution genomics data, will enable large-scale microbiome research. For a long time, gut *Bacteroides* have been used as representative commensal microorganisms to study host-microbiota interactions because they are easily cultured and genetically tractable.^159–161^ With the success of the CRISPR/Cas system in genome editing of eukaryotic cells, new CRISPR/Cas-based microbial gene-editing tools have emerged in recent years, targeting various members of the human gut bacterial communities.^22^^–162–164^ Of note, gut bacteria belonging to the Firmicutes/Clostridia are the most abundant species in the human gut, yet the development of genetic manipulation tools for these species has lagged behind. In recent work, an integrated pipeline with CRISPRi and Group II intron technologies was developed that enables gene editing in gut Firmicutes/Clostridia commensals, providing an avenue for genetic manipulation and functional studies for a complex microbiota.^165^ While bacterial genetics provides insight into microbial metabolic pathways, metabolomics directly measures the metabolic capacity of a given microorganism or community. MS-based metabolomic analysis can detect and quantify a large number of small molecules, but data analysis including feature detection and structure annotation remains challenging, especially for untargeted metabolomics.^166^ A recently developed open-access MS analysis platform, Global Natural Products Social Molecular Networking (GNPS), allows researchers to share and explore spectrometry data with enhanced annotations.^167^ Molecular networking connects mass spectra of molecules with similar fragment ion spectra, enabling meta-mass shift chemical profiling without knowledge of the chemical structures of related molecules.^168^ Using GNPS-based MS informatics, the researchers identified uncharacterized amino acid-conjugated bile acids, such as tyrosocholic acid, phenylalanocholic acid, and leucocholic acid, produced by the mammalian gut microbiota with agonistic activity on host FXR signaling.^33^ Once the chemical structure is revealed, researchers could apply reverse metabolomics to evaluate whether a given molecule is present in public untargeted metabolomics datasets and whether the molecule is associated with different biological parameters in each public dataset.^133^

In conclusion, this review summarizes recent advances in microbial transformation pathways and the immunomodulatory roles of gut lipid metabolites, such as short-chain fatty acids, long-chain fatty acids, and bile acids. Both host and diet-derived lipid precursors can be metabolized into different microbial lipid derivatives via distinct biotransformation pathways within the human gut microbiome. The resulting products modulate both the innate and adaptive branches of the host’s immune system by targeting different types of immune cells through receptor-dependent or -independent mechanisms. As new tools for studying the microbiome continue to emerge, we will gain a better understanding of the lipid chemistry and biotransformations of individual gut microbes at a functional level. This will allow us to further dissect the underlying mechanisms of microbial-immune interactions that control our health and disease.
